# Diffusion spectrum imaging in white matter microstructure in subjects with type 2 diabetes

**DOI:** 10.1371/journal.pone.0203271

**Published:** 2018-11-14

**Authors:** Qing Zhang, Yawen Xiao, Lin Lin, Jianlin Wu

**Affiliations:** Department of Radiology, Affiliated Zhongshan Hospital of Dalian University, Dalian, China; Chinese Academy of Sciences, CHINA

## Abstract

This study aimed to investigate the feasibility and clinical applicability of Diffusion Spectrum Imaging (DSI) for quantitative detection of white matter microstructural integrity. Twenty-seven patients with type 2 diabetes mellitus (T2DM; aged 60.6±7.6 years) and 21 healthy controls (HC; aged 56.1±7.8 years) underwent high-resolution T1-weighted imaging and DSI scanning. Cognitive function scores were obtained using such instruments as the Montreal Cognitive Assessment (MoCA). The fasting blood glucose, glycated hemoglobin, and total cholesterol (CHO) of T2DM were measured. The bilateral uncinate fasciculus and superior cingulum bundle were reconstructed by DSI tractography. Statistical analysis was performed using SPSS 21.0 software and P<0.05 was considered significant. Generalized fractional anisotropy (GFA) values were significantly decreased in the left uncinate fasciculus (t = −2.915, p = 0.005) and right superior cingulum bundle (t = −2.604, p = 0.012) in T2DM patients compared with the healthy controls (p<0.05). The MoCA (t = −3.339, p = 0.002) and CDT (t = −3.039, p = 0.004) scores of T2DM were significantly lower than those of healthy controls. Meanwhile, the GFA value of the right superior cingulum bundle was negatively associated with VFT score (r = −0.475, p = 0.012), and that of the right superior cingulum bundle was negatively associated with blood CHO level (r = −0.458, p = 0.016). DSI tractography is capable of evaluating the microstructural integrity of the white matter bundle in T2DM and is related to clinical cognitive scores and related biochemical indices; therefore, it can help to predict early white matter abnormalities in T2DM.

## Introduction

Previous findings have shown that type 2 diabetes mellitus (T2DM) could increase the risk of Alzheimer’s disease, stroke, cerebral white matter lesions, and cognitive dysfunction [1]. White matter abnormalities have been detected in some brain regions, including the frontal lobe, cingulum bundle (CB), uncinate fasciculus (UF), and anterior limb of the internal capsule. The bilateral frontal, temporal, and parietal lobe microstructural alterations were particularly remarkable [2–4]. The UF and CB are the main fibers interconnecting the above regions. Many crossing fibers exist in the frontal cortex [5], and multiple fiber orientations coexist within single voxels in the UF [6]; thus, the conventional diffusion tensor imaging (DTI) has limited descriptive value for this area. Diffusion spectrum imaging (DSI) is an advanced magnetic resonance imaging (MRI) technique that display crossing fibers and complex intravoxel fiber orientation distributions reliably and accurately [7]. Therefore, to investigate the feasibility and value of clinical application of DSI reconstruction, it was employed to detect and evaluate the microstructural integrity of the UF and superior CB and explore their relationship with performance on neuropsychological tests and related biochemical indicators.

## Material and methods

### Subjects

This study was approved by the Medical Ethics Committee of the Affiliated Zhongshan Hospital of Dalian University, and performed in accordance with the ethical guidelines of the Declaration of Helsinki. After complete description of the study to the subjects, written informed consent was obtained from each subject.

Twenty-seven patients with T2DM were selected from patient rolls of our hospital during August 2014 to January 2015. Written informed consent was obtained before the test. Participants with diabetes fulfilled the following inclusion criteria: (1) age range 45–70 years, (2) right-handed, (3) met the criteria of The American Diabetes Association “Clinical Practice Recommendations 2011”, (4) were free of hypoglycemia, and insulin treatment had been stopped by the scanning day. Twenty-one healthy control (HC) participants were selected and matched to patients by age, sex, handedness, and education. The exclusion criteria were (1) history of transient ischemic attacks or stroke within the past 2 years, (2) history of neurological disorders irrelevant to diabetes and affecting cognition, (3) evidence of structural abnormalities or brain white matter lesions (Age-Related White Matter Changes Scale score>1) on the structural MR scan, and (4) history of smoking, alcohol, or psychotropic substance abuse. In addition, fasting blood glucose, glycated hemoglobin, and total cholesterol (CHO) of T2DM were also obtained.

### Neuropsychological evaluations

All participants underwent the same neuropsychological test battery administered by a skilled doctor, including the Montreal Cognitive Assessment (MoCA), the Clock Drawing Test (CDT), and the verbal fluency test (VFT) to assess general cognitive function, executive function, and semantic verbal fluency, respectively.

### MRI data acquisition

Participants were scanned on a 3-Tesla (3T) MRI scanner (Vero, Siemens, Germany) with an 8-channel head coil (S1 Dataset). High-resolution T1-weighted images were acquired using a magnetization prepared 3D gradient-echo sequence, with repetition time (TR) = 1900 ms, echo time (TE) = 2.49 ms, flip angle = 9°, matrix size = 256×256, field of view (FOV) = 250 mm×250 mm, and slice thickness = 1 mm; the scanning time was 4 minutes 26 seconds. Axial T2-weighted images were acquired using a fast spin-echo sequence with 20 contiguous slices that covered the entire brain,with TR = 4000 ms, TE = 77 ms, flip angle = 150°, FOV = 250 mm×250 mm, and slice thickness = 5 mm; the scanning time was 56 seconds.

DSI was acquired using a pulsed gradient twice-refocused spin-echo diffusion echo-planar imaging sequence (TR/TE = 9600/130 ms, FOV = 200 mm×200 mm, matrix = 80×80, slice thickness = 2.5 mm). A total of 102 diffusion encoding directions were acquired within a half sphere of the q-space with maximum diffusion sensitivity (b_max_) 4000 s/mm^2^. The scan time for DSI acquisition was 16 minutes and 48 seconds.

### MR image processing and analysis

After DSI data acquisition, the diffusion probability density function (PDF) was obtained according to the Fourier relationship between the PDF and q-space signal [8]. The orientation distribution function (ODF) was computed by obtaining the second moment of the PDF along each of the 362 radial directions (6-fold tessellated icosahedron) [9]. Generalized fractional anisotropy (GFA), which is the DSI index equivalent to the DTI FA index, was computed for each voxel using the following formula: (standard deviation of the ODF)/(root mean square of the ODF) [10]. GFA ranges 0–1 and indicates the microstructural integrity of the white matter fiber tracts [11]. Higher GFA values illustrate greater structural integrity.

**Fig 1:**
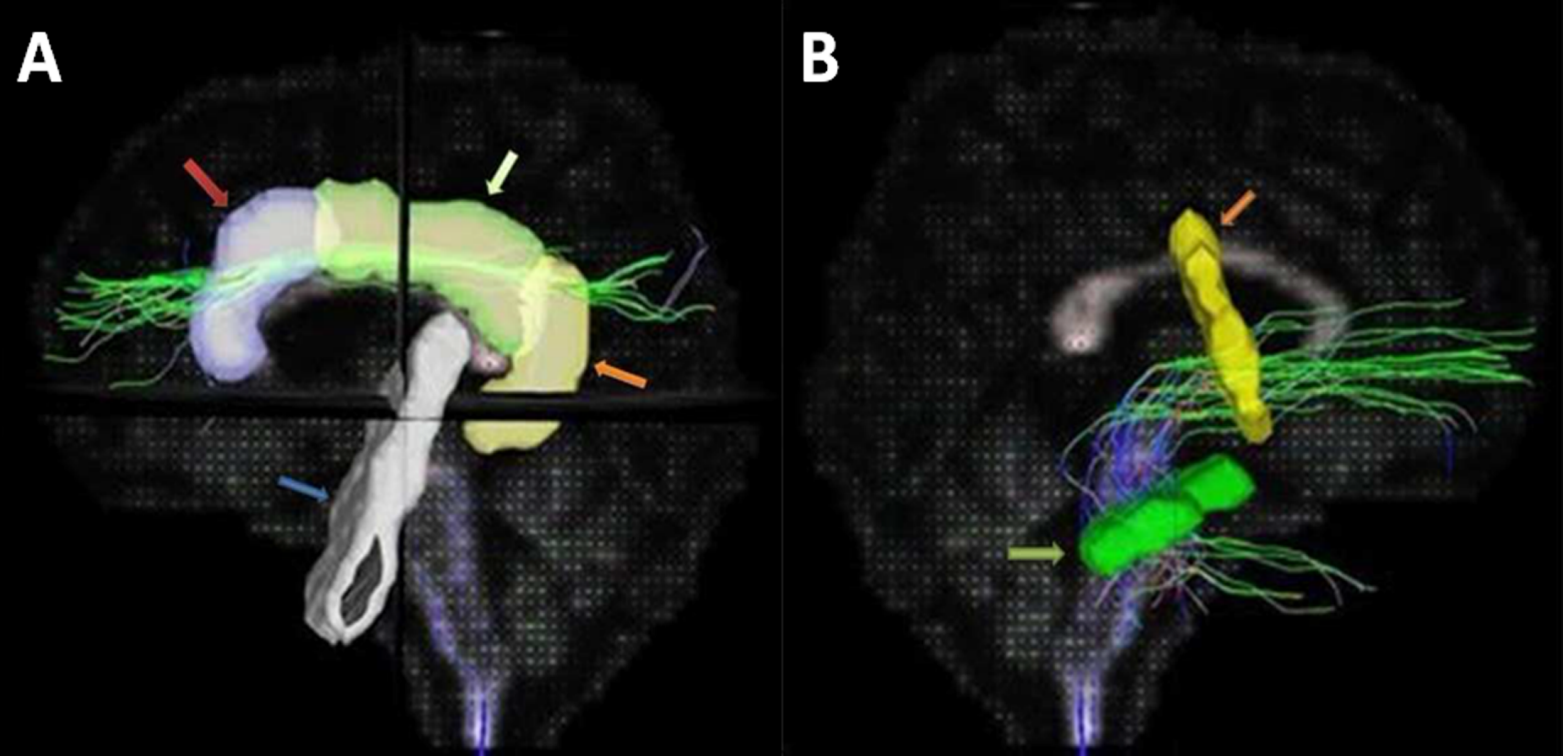
DSI tractography. A: ROIs of superior cingulum bundle: anterior cingulate gyrus (ROI1, blue area, red arrow), middle cingulate gyrus (ROI2, green area, yellow arrow), and posterior cingulate gyrus (ROI3, yellow area, orange arrow). The white area is an ROA between the genu and splenium of the corpus callosum. B: ROIs of uncinate fasciculus: axial slice after curvature to the temporal lobe (ROI1, green area, green arrow) and coronal slice in the frontal lobe next to the anterior horn of the lateral ventricle (ROI2, yellow area, orange arrow).

Tractography was performed according to the following procedure: (1) Image registration and normalization: we used the T1-weighted image as a reference and the b0 image (the null image in DSI) as the source image to perform a 3D affine transformation to coregister the diffusion images to the anatomical images. Then, the individual participants’ output images were wrapped to the Montreal Neurological Institute (MNI) template using the FLIRT, FSL software package (http://www.fmrib.ox.ac.uk/fsl/). (2) Region of interest (ROI) segmentation: ROIs under MNI space were selected using MRIcron software. For reconstruction of the UF, one ROI was placed on an axial slice after curvature to the temporal lobe and the other on a coronal slice in the frontal lobe next to the anterior horn of lateral ventricle [12]. For reconstruction of the superior CB, three “AND” ROIs were placed on the anterior, middle, and posterior cingulate gyri [5], and a region of avoid was placed between the genu and splenium of the corpus callosum to purify the bundle (Fig 1). (3) Generation of GFA map. After a brain GFA map was generated using the DSI Studio software package (http://dsi-studio.labsolver.org/), the coordinates of the MNI template ROIs were then mapped onto the individual participants’ brain GFA maps through an inverse transformation using a deformation matrix calculated using MATLAB and DSI Studio software. Then, a streamline-based fiber tracking algorithm was executed using DSI Studio to reconstruct the UF and superior CB. (4) Calculation of GFA: Tract-specific analysis was used to calculate the GFA values at each voxel and then calculate the mean GFA values of each pathway.

### Statistical analysis

Statistical comparisons were performed using SPSS version 21.0 (SPSS, Inc., Chicago, IL). The significance level for these statistical analyses was defined as P<0.05. Student’s t-tests were used to examine the between-group differences in demographic features, clinical indices, and continuous neurocognitive scores. χ^2^ tests were used for enumerative data, such as sex. DSI measures such as GFA values were compared between groups using two-sample t-tests and related to cognitive performance by Pearson correlation analysis with age and sex as covariates.

## Result

### Demographic features and neuropsychological scores

There were no significant between-group differences in sex distribution, age, or years of education (P>0.05). All measured neurocognitive scores (MoCA, CDT and VFT) were lower in patients with T2DM than HC. The differences in MoCA (t = −3.339, P = 0.002) and CDT (t = −3.039, P = 0.004) scores were statistically significant (Table 1).

**Table 1:** Between-group comparisons of demographic features and neuropsychological scores.

		DM	HC	t/*χ*^*2*^	P
Sex	M	9(33.3%)	5(23.9%)	0.519	0.471
	F	18(66.7%)	16(76.1%)		
T2DM duration(years)		13±7	-		
Education(years)		10±3	10±2	0.9	0.373
Age(years)		60.6±7.6	56.1±7.8	1.84	0.170
MoCA		22±3.45	24.76±1.76	−3.339	0.002*
CDT		2.74±0.66	3.29±0.56	−3.039	0.004*
VFT		44.44±7.54	48.81±8.54	−1.877	0.067

Note: Values are presented as count for sex and mean ± SD for age, T2DM duration, education, MoCA, CDT and VFT. M male, F female, MoCA Montreal Cognitive Assessment, CDT Clock Drawing Test,VFT Verbal Fluency Test. T2DM type 2 diabetes mellitus. HC healthy control

* P<0.05.

### Comparison of GFA value of UF and superior CB between groups

**Fig 2:**
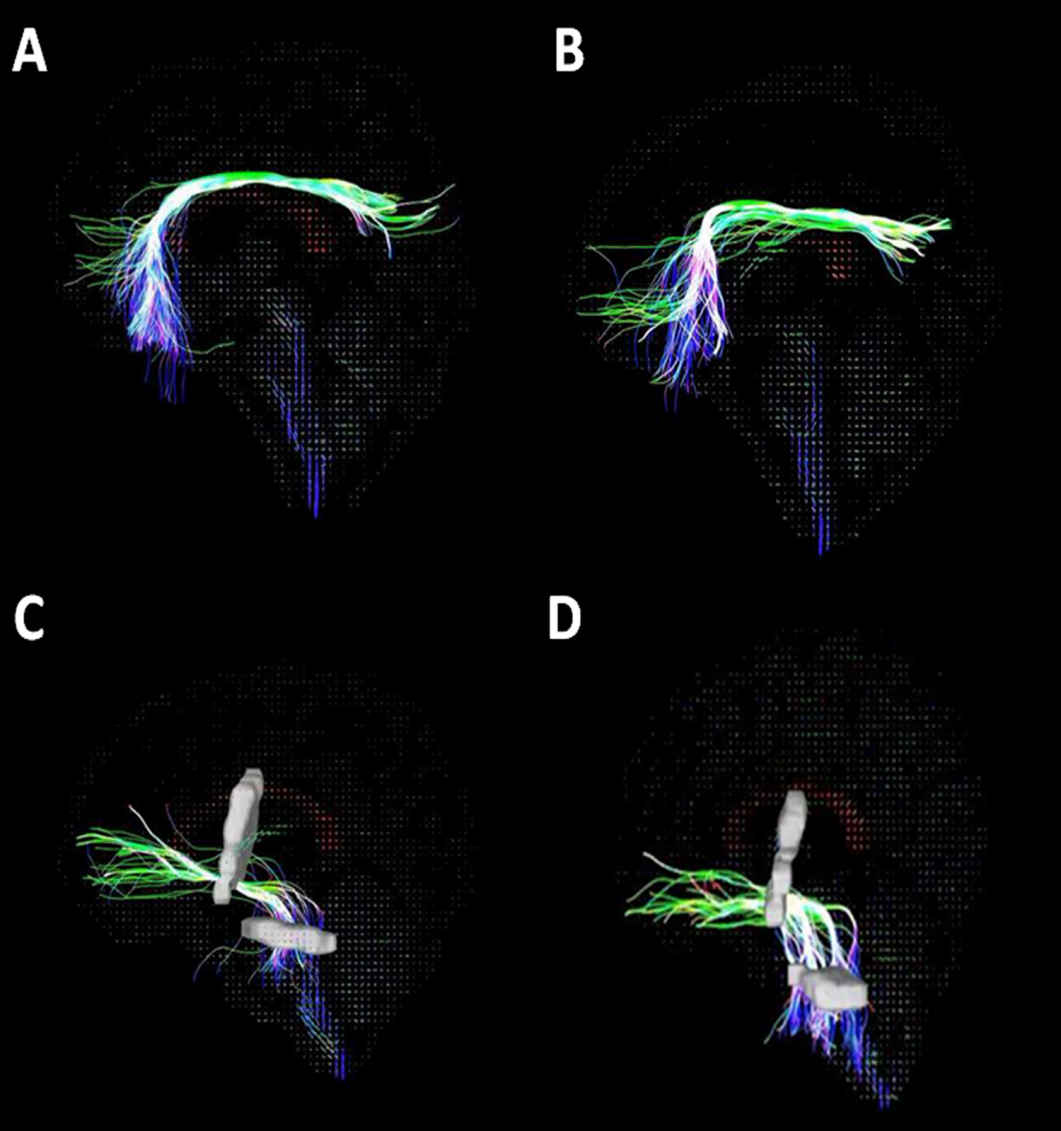
**A, B: Superior cingulum bundle tractography**. A: healthy control (male, age 51 years, MoCA score 24); B: T2DM (male, age 49 years, duration 12 years, MoCA score 17). **C, D: uncinate fasciculus tractography**. C: healthy control (male, age 60 years, MoCA score 27); D: T2DM (female, age 49 years, duration 4 years, MoCA score 22).

GFA values were significantly lower in the bilateral UF and right superior CB in patients with T2DM than HC (P<0.05). The differences in the left UF (t = −2.915, P = 0.005) and right superior CB (t = −2.604, P = 0.012) were statistically significant, as shown in intergroup analysis (Table 2 and Fig 2).

**Table 2:** Between-group comparisons of GFA values of bilateral UF and superior CB.

GFA	T2DM	HC	t	P
left UF	0.789±0.007	0.795±0.006	-2.915	0.005*
right UF	0.795±0.006	0.795±0.006	-0.294	0.770
left superior CB	0.817±0.009	0.816±0.013	0.326	0.746
right superior CB	0.809±0.01	0.816±0.008	-2.604	0.012*

Note: UF uncinate fasciculus, CB cingulum bundle

* P<0.05.

### Correlations between GFA values and clinical indices in T2DM

**Fig 3:**
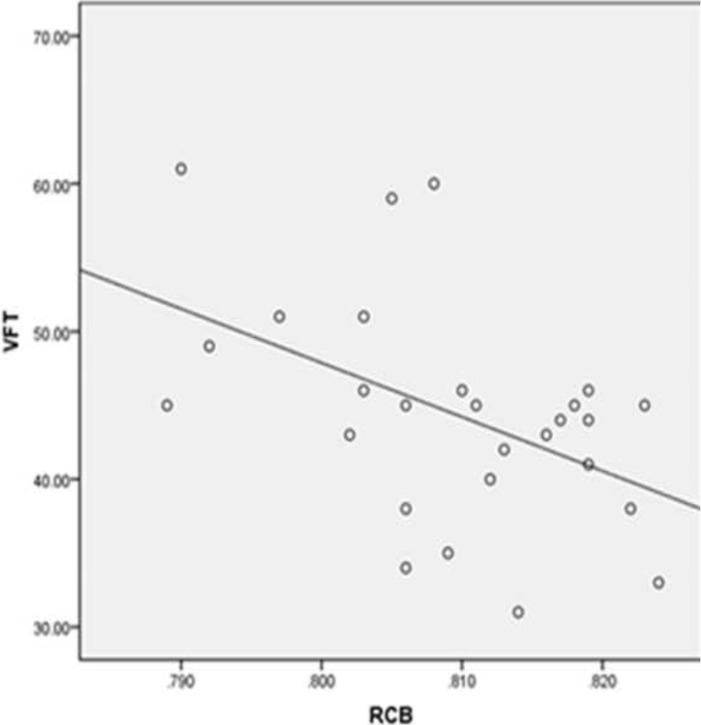
GFA value of the right superior cingulum. The bundle was negatively associated with VFT test. r = −0.475, P = 0.012.

**Fig 4:**
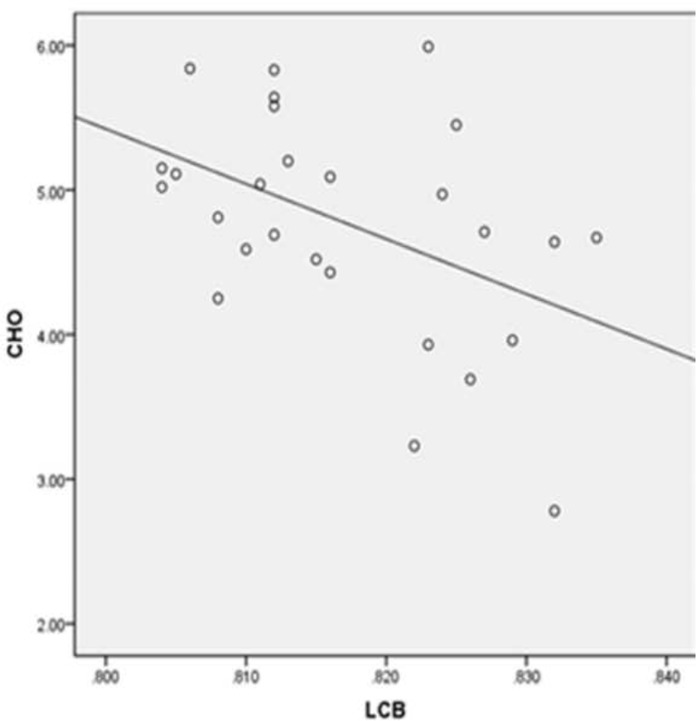
GFA value of the left superior cingulum. The bundle was negatively associated with CHO. r = −0.458, P = 0.016.

The GFA value in the right superior CB was negatively associated with VFT test score (r = −0.475, P = 0.012), and the GFA value in the left superior CB was negatively associated with blood CHO level (r = −0.458, P = 0.016) in T2DM, as shown in Pearson correlation analysis. However, there were no obvious correlations among the other variables (Table 3 and Figs 3 and 4).

**Table 3:** Correlational analysis of GFA values and related clinical indices in T2DM.

Clinical index	LU		RU		LCB		RCB	
	r	P	r	P	r	P	r	P
MoCA	-0.299	0.129	-0.154	0.444	-0.344	0.079	-0.273	0.168
CDT	-0.061	0.762	-0.049	0.810	-0.127	0.529	0.123	0.540
VFT	-0.051	0.802	0.123	0.542	-0.215	0.281	-0.475	0.012*
GLU	0.281	0.155	0.323	0.101	-0.045	0.825	-0.004	0.983
HbAlc	0.113	0.575	0.341	0.082	-0.064	0.750	-0.130	0.519
CHO	-0.122	0.544	0.136	0.499	-0.458	0.016*	-0.280	0.158

Note: GLU fasting blood glucose, HbAlc glycated hemoglobin, CHO total cholesterol. LU left uncinate fasciculus, RU right uncinate fasciculus, LCB left superior cingulum bundle, RCB right superior cingulum bundle

* P<0.05.

## Discussion

### Feasibility and value of DSI tractography for quantitative detection of the white matter bundle

Compared with DTI tractography, the unique mathematical models and algorithms of DSI were developed to trace crossing fibers and complex distributions of intravoxel fiber orientation reliably and accurately. Brain reconstruction via DSI has revealed neural connectivity, such as associative, association, and projection fibers. However, few previous reports focused on the UF and CB in patients with T2DM. DSI requires particularly long diffusion encoding gradients and high b values during MR signal evolution, leading to quite long acquisition time. We can shorten the total acquisition time by reducing the number of diffusion encoding gradient detections. Kuo LW [13] proposed optimum parameters for 3T MRI using 203 encoding gradient detections and a maximum b-value of 4000 s/mm^2^. However, total acquisition time under that protocol would last 30 minutes, and such long times are unacceptable for routine clinical application. In our study, a total of 102 DWI volumes were acquired within a half sphere of the q-space, where the maximum diffusion sensitivity value (b-value) corresponding to the sphere’s radius was 4000 s/mm^2^. The acquisition scheme could be modified to employ a half sphere rather than a full sphere because of the symmetric q-space sampling method. With this approach, the scanning time was reduced to approximately 16.5 minutes, and the data and results can satisfy clinical needs.

Previous studies have demonstrated that decreased cognitive function in patients with T2DM may be caused by abnormalities of the frontal, temporal, and parietal cortices; the UF and superior CB just are the main tracts connecting these brain regions, which participate in emotional processing, attention, memory, and other brain functions [14]. In this study, we found significantly decreased GFA values in the bilateral UF and right superior CB in patients with T2DM compared with HC (P<0.05), indicating significant degeneration of these neural fiber bundles in these patients, especially in the left UF and right superior CB. Because the participants in our study were all right-handed, the brain areas connected by the left UF may have more metabolic requirements for activation compared with those on the other side [15]. Thus, the left neural pathways were more likely show involvement in conditions of equivalent metabolic reserve, and the resulting GFA values in DSI were decreased. This finding is in accordance with Hoogenboom et al [2].The superior CB is comprised by anterior, middle, and posterior segments that are involved in emotion, perception, attention, memory, and motor tasks [16]. The left CB of normal, right-handed people has shown a more consistent direction of diffusion of water molecules along fiber tracts compared with the right CB [17]. Our study also demonstrated decreased microstructural integrity in the right superior CB in patients with T2DM, suggesting that the CB’s lower diffusion consistency may make hyperglycemia more likely to impair this neural fiber bundle. The significance of our study is to highlight DSI tractography as a sensitive, reliable tool for detecting structural changes in the UF and superior CB of patients with T2DM.

### Correlations between decreased GFA values and neurocognitive scores in T2DM

In our study, the neurocognitive scores (i.e., MoCA, CDT, and VFT) of patients with T2DM were lower than those of HC. This conforms to the results of the study of Zhang et al. [18], indicating that cognitive and memory-related decline caused by T2DM may be a critical determinant of degeneration of the structural integrity of the corresponding region.

We found that the GFA values of the right superior CB were negatively associated with VFT scores in T2DM (r = −0.475, P = 0.012), showing that the integrity of that hemisphere’s white matter fiber microstructure has no obvious effect on verbal fluency. Previous studies showed that a semantic fluency task activated the left temporal cortex [19], and the left medial temporal lobe has been associated with semantic fluency [20]. However, little information exists on the correlation between the impairment of right bundle with this task. Because there are widespread fiber connections and interactions between the hemispheres [21], and verbal memory is partially dependent on interhemispheric interactions [22], we infer that in T2DM, the left temporal lobe could also be activated under the semantic fluency task in the right CB’s impaired condition. Moreover, the seriousness of degeneration of the right brain region is associated with compensatory activation in response to interhemispheric interactions. As a result, subjects’ word production and fluency will increase accordingly. However, the detailed mechanism of this interaction needs to be explored further. We found no relationship between these bundles’ GFA values and MoCA and CDT scores, perhaps as the above tests are not designed to detect subcortical damage-related loss of function and hence not sensitive subtle cognitive changes correlated with microstructural changes in T2DM. In addition, the patient sample was relatively small, and as a result, some between-group differences may not have reached statistical significance.

### Correlations between decreased GFA values and biochemical indicators in T2DM

Our results showed that GFA values in the left superior CB were inversely associated with total CHO in patients with T2DM, indicating that hypercholesterolemia impacts the fibers’ microstructural integrity. Previous research found that high CHO levels could eliminate synaptic plasticity [23], and the deposition of amyloid-β protein neurotoxicity substance could increase the risk of nerve injury [24, 25], eventually resulting in cognitive impairment. Fang confirmed that high CHO aggravated hippocampal neuronal loss and glial cell proliferation in rats with an Alzheimer’s disease model. As the CB is the fiber tract connecting the hippocampi, high CHO results in fundamental pathological microstructural impairment of CB in T2DM. The participants were all right-handed; therefore, their left brain areas may had more metabolic demands than those on the right. Thus, the left neural pathways were most likely to show involvement under equivalent conditions.

Research has suggested that abnormalities in microstructural integrity were inversely correlated with fasting blood glucose and glycosylated hemoglobin [2]. However, our study showed no correlation between decreased microstructural integrity of the UF or superior CB with clinical indices such as fasting glucose levels. We supposed that longer T2DM duration (>4 years) and treatment to control T2DM (>1 year) would lead blood glucose parameters like fasting blood glucose and glycosylated hemoglobin to remain within a relatively stable range. On the contrary, the untreated lipid profile parameters of these patients with T2DM better reflect actual pathophysiological status than the above glucose parameters.

This study had several limitations. First, the patient sample size was modest, which limits the study’s statistical power. Second, because the ROIs in our study were delineated manually, there was inevitable slight anatomical structural positional deviation that persisted after spatial normalization. In addition, the participants received therapy to control their blood glucose levels, which may influence certain results. In conclusion, this study demonstrated microstructural abnormalities in related white matter tracts in T2DM, which were associated with neuropsychological scores and biochemical indices. Assessment of microstructural changes with DSI tract-specific analysis is more sensitive than classical MRI markers and may be able to predict early white matter abnormalities in T2DM.

## Supporting information

S1 Dataset One of the MR images from T2DM patients group. (RAR)

## References

[1] Benedict C, Brooks SJ, Kullberg J, Burgos J, Kempton MJ, Nordenskjöld R, et al Impaired insulin sensitivity as indexed by the HOMA score is associated with deficits in verbal fluency and temporal lobe gray matter volume in the elderly. Diabetes Care. 2012;35(3): 488–494. 10.2337/dc11-2075 22301128 PMC3322700

[2] Hoogenboom WS, Marder TJ, Flores VL, Huisman S, Eaton HP, Schneiderman JS, et al Cerebral white matter integrity and resting-state functional connectivity in middle-aged patients with type 2 diabetes. Diabetes. 2014;63(2): 728–738. 10.2337/db13-1219 24203723 PMC3900542

[3] Zhang A, Ajilore O, Zhan L, Gadelkarim J, Korthauer L, Yang S, et al White matter tract integrity of anterior limb of internal capsule in major depression and type 2 diabetes. Neuropsychopharmacology. 2013;38(8): 1451–1459. 10.1038/npp.2013.41 23389692 PMC3682138

[4] Voineskos AN, Rajji TK, Lobaugh NJ, Miranda D, Shenton ME, Kennedy JL, et al Age-related decline in white matter tract integrity and cognitive performance: a DTI tractography and structural equation modeling study. Neurobiol Aging. 2012;33(1): 21–34. 10.1016/j.neurobiolaging.2010.02.009 20363050 PMC2945445

[5] Lin YC, Shih YC, Tseng WY, Chu YH, Wu MT, Chen TF, et al Cingulum Correlates of Cognitive Functions in Patients with Mild Cognitive Impairment and Early Alzheimer’s Disease: A Diffusion Spectrum Imaging Study. Brain Topogr. 2014;27(3): 393–402. 10.1007/s10548-013-0346-2 24414091

[6] Vos SB, Jones DK, Jeurissen B, Viergever MA, Leemans A. The influence of complex white matter architecture on the mean diffusivity in diffusion tensor MRI of the human brain. Neuroimage. 2012;59(3): 2208–2216. 10.1016/j.neuroimage.2011.09.086 22005591 PMC7613439

[7] Wedeen VJ, Wang RP, Schmahmann JD, Benner T, Tseng WY, Dai G, et al Diffusion spectrum magnetic resonance imaging (DSI) tractography of crossing fibers. NeuroImage. 2008;41(4): 1267–1277. 10.1016/j.neuroimage.2008.03.036 18495497

[8] Callaghan P. Principles of Nuclear Magnetic Resonance Microscopy. Clarendon Press: Oxford 1991.

[9] Wedeen VJ, Hagmann P, Tseng WY, Reese TG, Weisskoff RM. Mapping complex tissue architecture with diffusion spectrum magnetic resonance imaging. Magn Reson Med. 2005;54(6): 1377–1386. 10.1002/mrm.20642 16247738

[10] Tuch DS. Q-ball imaging. Magn Reson Med. 2004;52(3): 1358–1372. 15562495 10.1002/mrm.20279

[11] Fritzsche KH, Laun FB, Meinzer HP, Stieltjes B. Opportunities and pitfalls in the quantification of fiber integrity: what can we gain from Q-ball imaging? NeuroImage. 2010;51(1): 242–251. 10.1016/j.neuroimage.2010.02.007 20149879

[12] Fujie S, Namiki C, Nishi H, Yamada M, Miyata J, Sakata D, et al The Role of the Uncinate Fasciculus in Memory and Emotional Recognition in Amnestic Mild Cognitive Impairment. Dement Geriatr Cogn Disord. 2008;26(5): 432–439. 10.1159/000165381 18957848

[13] Kuo LW, Chen JH, Wedeen VJ, Tseng WY. Optimization of diffusion spectrum imaging and q-ball imaging on clinical MRI system. Neuroimage. 2008;41(1):7–18. 10.1016/j.neuroimage.2008.02.016 18387822

[14] Kuo LW, Chiang WY, Yeh FC, Wedeen VJ, Tseng WY. Diffusion spectrum MRI using body-centered-cubic and half-sphere sampling Schemes. J Neurosci Methods. 2013;212(1): 143–155. 10.1016/j.jneumeth.2012.09.028 23059492

[15] Gong G, Jiang T, Zhu C, Zang Y, He Y, Xie S, et al Side and handedness effects on the cingulum from diffusion tensor imaging. Neuroreport. 2005;16(15): 1701–1705. 16189481 10.1097/01.wnr.0000183327.98370.6a

[16] Shipton OA, El-Gaby M, Apergis-Schoute J, Deisseroth K, Bannerman DM, Paulsen O, et al Left-right dissociation of hippocampal memory processes in mice. Proc Natl Acad Sci U S A. 2014 10 21;111(42): 15238–15243. 10.1073/pnas.1405648111 25246561 PMC4210314

[17] Beckmann M, Johansen-Berg H, Rushworth MF. Connectivity-based parcellation of human cingulate cortex and its relation to functional specialization. J Neurosci. 2009;29(4): 1175–1190. 10.1523/JNEUROSCI.3328-08.2009 19176826 PMC6665147

[18] Zhang YW, Zhang JQ, Liu C, Wei P, Zhang X, Yuan QY, et al Memory dysfunction in type 2 diabetes mellitus correlates with reduced hippocampal CA1 and subiculum volumes. Chin Med J (Engl). 2015;128(4): 465–471. 25673447 10.4103/0366-6999.151082 PMC4836248

[19] Mummery CJ, Patterson K, Hodges JR, Wise RJ. Generating 'tiger' as an animal name or a word beginning with T: differences in brain activation. Proc Biol Sci. 1996;263(1373):989–995. 10.1098/rspb.1996.0146 8805836

[20] Pihlajamäki M, Tanila H, Hänninen T, Könönen M, Laakso M, Partanen K, et al Verbal fluency activates the left medial temporal lobe: a functional magnetic resonance imaging study. Ann Neurol. 2000; 47(4): 470–476. 10762158

[21] Stuss DT, Alexander MP, Hamer L, Palumbo C, Dempster R, Binns M, et al The effects of focal anterior and posterior brain lesions on verbal fluency. J Inter N europsychol Soc. 1998; 4(3): 265–278. 9623001

[22] Christman SD, Propper RE. Superior episodic memory is associated with interhemispheric processing. Neuropsychology. 2001;15 (4): 607–616. 11761050 10.1037//0894-4105.15.4.607

[23] Pfrieger FW. Role of cholesterol in synapse formation and function. Biochem Biophys Acta. 2003;1610(2):271–280. 12648780 10.1016/s0005-2736(03)00024-5

[24] Lu J, Zheng YL, Wu DM, Sun DX, Shan Q, Fan SH. Trace amounts of copper induce neurotoxicity in the cholesterol-fed mice through apoptosis. FEBS Lett. 2006;580(28–29): 6730–6740. 10.1016/j.febslet.2006.10.072 17134702

[25] Gonzalo-Ruiz A, Pérez JL, Sanz JM, Geula C, Arévalo J. Effects of lipids and aging on the neurotoxicity and neuronal loss caused by intracerebral injections of the amyloid-beta peptide in the rat. Exp Neurol. 2006;197(1): 41–55. 10.1016/j.expneurol.2005.06.008 16045911

